# PolyC-binding proteins enhance expression of the CDK2 cell cycle regulatory protein *via* alternative splicing

**DOI:** 10.1093/nar/gkx1255

**Published:** 2017-12-14

**Authors:** Xinjun Ji, Jesse Humenik, Daphne Yang, Stephen A Liebhaber

**Affiliations:** 1 Department of Genetics, Perelman School of Medicine, University of Pennsylvania, Philadelphia, PA 19104, USA; 2 Department of Medicine, Perelman School of Medicine, University of Pennsylvania, Philadelphia, PA 19104, USA

## Abstract

The PolyC binding proteins (PCBPs) impact alternative splicing of a subset of mammalian genes that are enriched in basic cellular functions. Here, we focus our analysis on PCBP-controlled cassette exon-splicing within the cell cycle control regulator cyclin-dependent kinase-2 (CDK2) transcript. We demonstrate that PCBP binding to a C-rich polypyrimidine tract (PPT) preceding exon 5 of the CDK2 transcript enhances cassette exon inclusion. This splice enhancement is U2AF65-independent and predominantly reflects actions of the PCBP1 isoform. Remarkably, PCBPs’ control of CDK2 ex5 splicing has evolved subsequent to mammalian divergence *via* conversion of constitutive exon 5 inclusion in the mouse CDK2 transcript to PCBP-responsive exon 5 alternative splicing in humans. Importantly, exclusion of exon 5 from the hCDK2 transcript dramatically represses the expression of CDK2 protein with a corresponding perturbation in cell cycle kinetics. These data highlight a recently evolved post-transcriptional pathway in primate species with the potential to modulate cell cycle control.

## INTRODUCTION

RNA splicing is a highly dynamic process that is regulated by a broad array of RNA binding proteins (RBP). Many of these RBPs constitute core components of mammalian spliceosome complexes (1) or interact with these complexes to modulate assembly of the splicing machinery (2). The activity of a splice acceptor site located 5′ to an exon is a frequent target of splicing control and its activity is often determined by the efficiency with which RNP complexes assemble at the polypyrimidine tract (PPT) located immediately 5′ of the AG splice acceptor dinucleotide. This PPT consists of a loosely defined U-rich sequence with interspersed C residues (2–6). The activity of a splice acceptor can be specifically modulated by alterations in the efficiency with which the canonical PPT binding protein, U2AF65, binds and nucleates U2 spliceosome assembly. Interactions of RNA binding proteins to a PPT can alter initial U2AF65 binding, splicing complex assembly, and/or provide alternative pathways to activate splice acceptor functions (3,6–8).

The PCBP proteins (also referred to as αCPs and hnRNPEs) comprise a family of RNA binding proteins with a strong binding specificity to C-rich polypyrimidine motifs (9–11). These proteins are encoded at four dispersed loci (12). In a recent study, we defined a global impact of the two most abundant and widely expressed PCBPs, PCBP1 and PCBP2, on alternative splicing (AS) of cassette exons (13). Analysis of cassette exons enhanced by PCBP1 and PCBP2 (ie., exons whose splicing was negatively impacted by acute depletion of PCBPs) revealed a marked enrichment for C-rich PPTs 5′ to the corresponding exons. Further analyses supported a model in which PCBPs and U2AF65 can activate distinct subsets of splice acceptors *via* binding to C-rich and U-rich PPTs, respectively. These data supported a model in which PCBPs play a pivotal role in gene regulation (13) by activating/modulating a distinct, U2AF65 independent, splicing pathway (2).

Transcriptome-wide analysis of cassette exons whose splicing is enhanced by PCBPs1/2 revealed enrichment for a subset of transcripts that encode proteins involved in basic cellular functions including cell death and survival, cellular growth and proliferation, and cell cycle control (13). Prominent among these targets of PCBP splicing control is the transcript encoding CDK2 (13). The cell cycle is regulated by the concerted actions of cyclin-dependent kinases (CDKs), cyclins, and CDK inhibitors (14–16). The CDKs are activated through pairing with specific cyclin partners (14,17). During early G1, CDK2 pairing with cyclin E promotes entry into the S phase of cell division cycle. CDK2 then switches to partner with cyclin A to drive the cell though S phase (15–17). The activities of the CDKs are tightly controlled and CDK activities can be repressed by a set of inhibitors including P21 (18). Dysregulation of the cell cycle and loss of proliferative controls is intimately linked to cell transformation and cancer.

In this report, we focus our studies on cassette exon splicing within the gene transcript encoding the cell cycle regulator, CDK2. We find that splicing of CDK2 exon 5 is positively regulated by PCBPs and that this control is mediated *via* interactions of PCBPs with a C-rich PPT splice acceptor 5′ to exon 5. Exclusion of exon 5 from the CDK2 mRNA markedly represses CDK2 protein expression and impacts on cell cycle control. Of further interest is the observation that CDK2 exon 5 has been converted during recent mammalian evolution from a constitutive exon (in the mouse) to an alternatively spliced cassette exon (in humans). This alteration in splicing control in primate lineage is linked to a corresponding acquisition of PCBP interaction with the PPT of exon 5 in the human CDK2 transcript. These data support a model in which PCBP proteins support the splicing of a defined subset of cassette exons by directly binding to cytosine-rich PPTs and that this PCBP-controlled pathway of post-transcriptional control can play a prominent role in the regulation of cell growth and proliferation.

## MATERIALS AND METHODS

### Cell culture and siRNA/shRNA transfection

**Figure 1.**
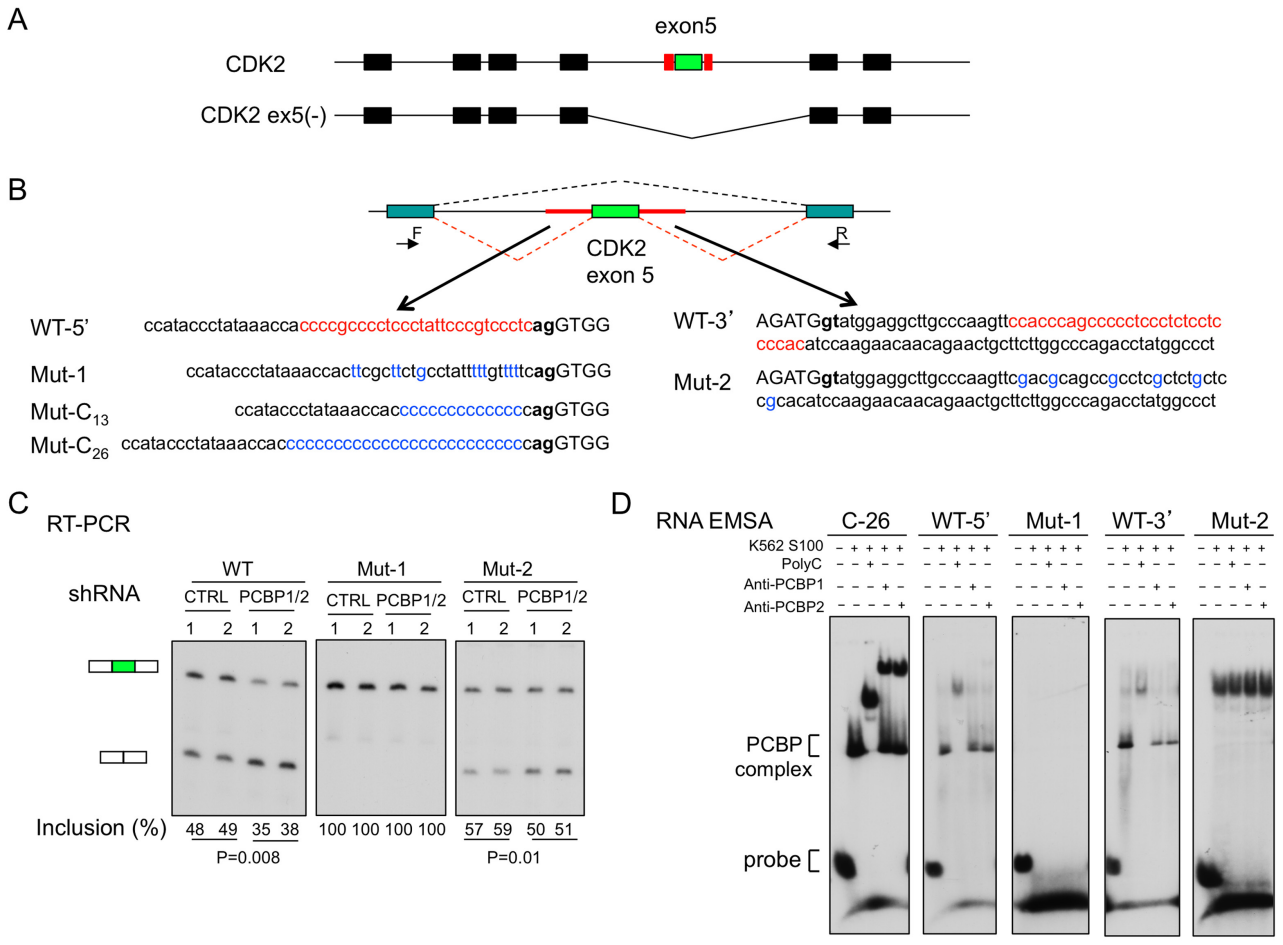
Minigene analysis links the C-rich composition of the exon 5 splice acceptor PPT to alternative splicing of the CDK2 transcript. (**A**) Diagram of the alternatively spliced CDK2 gene transcript. The top diagram shows the seven exons (boxes) included in the full length CKD2 mRNA (CDK2). The second diagram shows the exonic structure of the alternatively spliced CDK2 ex5(–) variant mRNA. The alternatively spliced exon 5 (green exon) is flanked 5′ and 3′ by C-rich motifs (red boxes). (**B**) CDK2 ex5 minigenes with sequence alterations in the C-rich intronic segments flanking exon 5. **Top:** CDK2 ex5 (green exon) along with its flanking contiguous C-rich intron sequences (red lines) was inserted into a minigene splicing vector. The forward (F) and reverse (R) primers used for splice analysis of exon 5 inclusion are shown. **Bottom:** Base substitutions within the C-rich sequences flanking the CDK2 ex5 are highlighted (red → blue fonts). Exonic sequences are in upper case and intronic sequences in lower case. The acceptor dinucleotide, **ag**, and the splice donor dinucleotide, **gt**, are bolded. (**C**) Splicing analysis. Each minigene plasmid (as in B, above) was co-transfected into K562 cells along with plasmids expressing either shRNA that co-depletes PCBP1 and PCBP2 (PCBP1/2) or a control shRNA. The splicing efficiency of exon 5 was assessed by RT-PCR (primers as in B, above) after a 3-day incubation. The % exon 5 inclusion values (Inclusion (%)) were calculated (see Methods) and are shown below the respective lanes. P values are shown. In each study the number of experimental biological repeats was 2 (*n* = 2). (**D**) RNA EMSA analyses of mutant splice sites. A C_26_ homopolymer served as a positive control for formation of the PCBP/RNA complex (PCBP complex). Super-shifts using antibodies to PCBP1 or PCBP2 confirmed the composition of the indicated PCBP complex.

K562 cells were cultured in RPMI 1640 medium, mouse erythroleukemia (MEL) cells were cultured in minimal essential medium (MEM) supplemented with 10% fetal bovine serum (HyClone) and antibiotic/antimycotic (Gibco) at 37°C in a 5% CO_2_ incubator. K562 cells were transfected with indicated siRNAs or shRNAs using Nucleofector V (Amaxa) as previously described (19). The two PCBP1/2 co-depletion siRNAs, siRNAs to U2AF65, siRNAs to PCBP1, siRNAs to PCBP2, negative control siRNA (medium GC content), and recombinant PCBP proteins were as described (13). siRNAs to PTBP1 (HSS143520, HSS143518, HSS183787), siRNAs to hnRNPK (HSS179311, HSS179312, HSS179313), and siRNAs to RBM39 (HSS145210, HSS145212, HSS190435) were purchased from Invitrogen. In Figure 6, we used cocktail of PCBP1siRNAs (composed of the three distinct PCBP1 siRNAs) or cocktail of PCBP2 siRNAs (composed of the three distinct PCBP2 siRNAs). In Figure 1C, we used PCBP1/2 shRNA converted from PCBP(1/2)-2 siRNA and its parental vector pGFP-V-RS as control (Origene Technologies, Inc.). MEL cells were stably transduced with 3 PCBP1 shRNAs (1–1:CCGCTAAGAATTTAAAGAA, 1–2: ACCGAGTGTGTGAAGCAGA, 1–3: CCTACCAATGCCATCTTTA, together with a control shRNA (Luciferase, CGCCTGAAGTCTCTGATTA).

### Western blot analysis

Total cellular extracts were prepared from the Trizol fraction of transfected K562 cells according to manufacturer's instruction. Extracts were separated on NuPAGE gel and electroblotted to nitrocellulose membranes (Protran BA 85; Schleicher & Schuell) for 1 h at 150 mA in transfer buffer (20 mM Tris, 150 mM glycine, 20% methanol) using a Semi-phor transfer apparatus (Hoefer). The membranes were blocked in 1% nonfat milk in 1× PBS for 1 h at room temperature, followed by three hours with primary antisera. Signals were visualized by ECL (ECL reagents; Boehringer Mannheim) or a LICOR Odyssey CLx Imaging platform with a fluorophore conjugated secondary antibody (IRDye800CW anti-rabbit, #925–32213, LI-COR, Lincoln, NE). Primary rabbit antibodies to CDK2 were purchased from Bethyl (A301–812A) and Abcam (ab32147). Anti-RBM39 antibody was purchased from Bethyl (A300–291A). Anti-U2AF65 antibody, a mouse monoclonal (U4758), was purchased from Sigma. Anti-hnRNPK antibody was purchased from Bethyl (A-300–674A). Anti-PTB antibody was purchased from Invitrogen (32–4800). Anti-Flag antibody was purchased from Agilent (anti-Flag, M2). Antibodies for PCBP1 and PCBP2 were as described (9). HRP-labeled secondary antibodies (Amersham) were used as detailed by the supplier. Donkey anti-rabbit immunoglobulin G (IgG)-horseradish peroxidase and sheep anti-mouse immunoglobulin G (IgG)-horseradish peroxidase (HRP) secondary antibodies were used at a 1:5000 dilution (Amersham).

### RNA EMSA

RNA oligonucleotides (Table 1) were synthesized and 5′-end labeled using T4 polynucleotide kinase (NEB, Beverly, MA) and [γ-^32^P]ATP (Amersham). All labeled oligonucleotides were gel purified on 12% denaturing gels prior to use. Electrophoretic mobility shift assays (EMSA) were carried out as described (9,13,19) with minor modifications. 5 ng of each probe (∼20 000 cpm) was mixed with 30 μg of K562 S100 extract, The incubation comprised 20 μl of binding buffer (10 mM Tris–HCl [pH 7.4], 150 mM KCl, 1.5 mM MgCl_2_, and 0.5 mM dithiothreitol) at room temperature for 20 min. One microliter of heparin (50 mg/ml) was added to each reaction mixture for 10 min prior to loading. Samples were resolved on a nondenaturing 5% polyacrylamide gel.

**Table 1.** RNA Oligos for RNA EMSA assay

CDK2-WT-5′	accccgccccucccuauucccgucccucag
CDK2-Mut1	acuucgcuucugccuauuuuuguuuuucag
CDK2-Mut2	cgacgcagccgccucgcucugcuccgcaca
CDK2-Mut3	acuucgcuucugccuauucccgucccucag
CDK2-Mut4	acuucgcuucugccuauuuccguuccucag
CDK2-WT-3′	ccacccagcccccucccucuccuccccaca
CDK2-13C	acccuauaaaccacccccccccccccccag
CDK2-26C	acccccccccccccccccccccccccccag
mCDK2-WT	uguaagcccuguugucuucuuguuccccag

### RT-PCR

RNAs were treated with amplification grade DNase I (Invitrogen) and then reverse transcribed using oligo-dT, Moloney murine leukemia virus reverse transcriptase (Promega), and 1 x Moloney murine leukemia virus reverse transcription (RT) buffer (Promega) according to manufacturer's instructions. After incubation at 37°C for 1 h, the samples were used as a template for PCR. The forward primer (20 pmol) was end labeled by incubation with [γ^32^ P]ATP and T4 PNK kinase (New England Biolabs). The PCRs included 1 μl of the RT product, 0.2 mM dNTPs, 1.5 mM MgCl_2_, 1 pmol of the labeled primer, 20 pmol of each primer, 0.25 U of AmpliTaq (Perkin Elmer), and 1 x PCR buffer II (Perkin Elmer) in a 25-μl reaction. The number of PCR cycles in each study was adjusted to the primers used and originating RNA concentrations so as to be in linear range. Samples were visualized by 6% denaturing polyacrylamide gel electrophoresis (PAGE) and quantified by the phosphorImager (ImageQuant; Molecular Dynamics). Alternative splicing efficiency was determined by calculating the inclusion level (%) of the cassette exon in all isoforms. RT-PCR primers: for human CDK2, (forward) 5′-GCTTTTGGAGTCCCTGTTCG-3′ and (reverse) 5′-GGTCCCCAGAGTCCGAAAGA-3′; for mouse CDK2, (1-F) 5′-AGCCCCAGAACCTGCTTATC-3′, (1-R) 5′-CCTTGTGATGCAGCCACTTC-3′, (2F) 5′-TTGGAGTCCCTGTCCGAACT-3′, (2-R) 5′-CCCCAGAGTCCGAAAGATCC-3′. For real-time RT-PCR, the primers are: CDK2-F: 5′-CAGGATGTGACCAAGCCAGT-3′, CDK2-R: 5′-TGAGTCCAAATAGCCCAAGG-3′.

### Minigene analysis of alternative 5′ and 3′ splice sites

WT and mutant CDK2 minigenes were cloned into the pI-11(-H3)-PL adenovirus based splicing minigene plasmid as described (13). The minigene inserts were PCR amplified with primers (forward, 5′-TGGAAATCAGTGGGAGGGGA-3′ and (reverse, 5′-TTTCCCACCATTCCCCTTGG-3′) and cloned onto the vector at the XbaI and XhoI cloning sites. Co-transfections of minigene plasmids and plasmids encoding defined recombinant proteins were performed as described (13). RT-PCR was done with common SP6 and T7 primers.

### CRISPR-Cas9 mediated mutation of the CDK2 locus in K562 cells and clonal selection

Four guide RNAs (sgRNA) for CDK2 exon 5 were designed, two upstream and two downstream of CDK2 exon 5 (see Figure 5 for detail). These sgRNAs were cloned into the pSpCas9(BB)-2A-PURO (PX459; Addgene) vector according to established protocol (20). These sgRNA vectors were combined and electroporated into K562 cells using Nucleofector V (Amaxa) as previously described (19). After 72 h, cells were selected by Puromycin (1 μg/ml) for one week. The selected cells were diluted and plated on 96-well plates for clonal isolation. These cell clones were screened for expected deletion by PCR (forward primer: 5′-TCCCTGTTCGTACTTACACCC-3′; reverse primer: 5′-CACCATTCCCCTTGGTGCTA-3′). Positive and control single clones were selected and confirmed by PCR amplification, RT-PCR, and DNA sequencing. The defined structures of these CDK2(-ex5) clones are as follows. In all cases the noted deletions obviate inclusion of exon 5 into the mature CDK2 mRNA. Clone #21 is a compound heterozygote: in one allele the entire exon 5 along with some adjacent flanking intronic sequences have been deleted, and the other allele has a deletion extending from 14 bp 5′to the splice acceptor site to the first 26 bp of exon 5. Clone #67 is homogenous for deletion extending from 24 bp 5′ to the splice acceptor site through to the first nucleotide of exon 5. Clone #68 is homozygous for a deletion extending from 21 bp 5′ to the splice acceptor through to the first two nucleotides of exon 5 of CDK2 gene. Clone #84 is a compound heterozygote: one allele is missing the entire exon 5 with some flanking intronic sequences (as in clone #21) while the other allele has a deletion extending from of 11 bp 5′ to the splice acceptor splicing through to include 12 bp from the 5′ terminus of exon 5.

### Cell cycle analysis

Control and exon 5(–) K562 cell clones were cultured and fixed with 80% ethanol and stored at –20°C. Cells were then washed with PBS, followed by a single wash in stain buffer (BD, 554656), and then incubated for 15 min with propidium iodide /RNase staining buffer (BD, 550825) at room temperature. The samples were analyzed by flow cytometry on BD LSR II and FlowJo software.

### Statistics

Statistical significance (*P* values) was determined using one-tailed, unpaired Student's *t* test. ^∗^*P* < 0.05, ^∗∗^*P* < 0.01, ^∗∗∗^*P* < 0.001, n.s.: not significant.

## RESULTS

### The C-rich poly-pyrimidine tract 5′ to CDK2 exon 5 plays an essential role in cassette exon splicing control

We have previously reported that the polyC binding proteins, PCBPs 1 and 2 (also known as αCPs and hnRNPEs), enhance the splicing of a subset of cassette exons (13). These data were generated by transcriptome analyses of the ENCODE Tier 1 human hematopoetic cell line, K562, acutely depleted of these two RNA binding proteins. These studies further revealed that exons enhanced by PCBPs are preceded at a high frequency by PPTs that are markedly enriched for C residues. One of the most strongly impacted of these PCBP-enhanced AS events is exon 5 of the transcript encoding the cell cycle regulatory kinase, CDK2. Analysis of native transcripts in K562 cells revealed that acute depletion of PCBPs1/2 resulted in a decrease from 87% to 48% in exon 5 inclusion in CDK2 mRNA (13). Exon 5 of the CDK2 transcript is flanked 5′ and 3′ by C-rich motifs (Figure 1A, red boxes). Here we explore in detail this AS event with the goal of extending our understanding of the *cis* and *trans* determinants of this regulatory pathway and linking these controls to a critical cell function.

To facilitate analyses, the CDK2 ex5 along with its flanking intronic C-rich motifs was transferred to a splice-reporter minigene (Figure 1B). The CDK2 ex5 minigene was transfected into K562 cells along with a shRNA co-targeting the two abundant and ubiquitously expressed PCBP isoforms, PCBP1 and PCBP2. Analysis of cellular RNA three days post-transfection revealed that PCBP1/2 co-depletion repressed CDK2 ex5 splicing to a significant extent when compared to scrambled shRNA controls (Figure 1C, WT panel). This result in the minigene context parallels the negative impact of PCBP1/2 co-depletion on exon 5 inclusion in the native CDK2 transcript (13). These data support the enhancement of exon 5 inclusion by PCBPs and serve to validate the CDK2 ex5 minigene model for subsequent analysis of this effect.

CDK2 ex5 is flanked 5′ and 3′ by C-rich motifs. The 5′ C-rich region maps to the exon 5 splice acceptor PPT and the 3′ C-rich region is located 20 nt 3′ to the intron 5 splice donor site (Figure 1B, red font). The individual contribution of each of the two C-rich regions to exon 5 splicing was investigated by altering their C-rich compositions. Although disruption of the C-rich region 3′ to exon 5 (Figure 1C, Mut-2) increased the baseline level of exon 5 inclusion, the negative impact of PCBP1/2 depletion on exon 5 splicing was maintained. This result suggests that the C-rich motif 3′ to CDK2 ex5, while contributing in some way to baseline splicing efficiency, does not play an appreciable role in the PCBP-mediated splicing control. With this result in hand we focused attention on the role of the C-rich PPT 5′ to CDK2 ex5.

To assess the impact of the C-rich sequences 5′ to exon 5 on cassette splicing, the sequence of the PPT was modified from C-rich to U-rich (canonical PPT) (Figure 1B, Mut-1). RNA EMSAs confirmed that this C → U sequence shift resulted in loss of PCBP binding *in vitro* (Figure 1D). RNA analysis of the transfected K562 cells revealed that this shift to a U-rich PPT was paralleled by a dramatic increase in the baseline levels of exon inclusion: 48% in the WT increased to 100% in the Mut-1. Importantly, the PCBP1/2 co-depletion failed to repress exon 5 splicing in the Mut-1 transcript (Figure 1C). When the WT PPT site was replaced with PPT of intermediate C/U composition, we again observed increased exon 5 inclusion and a failure of PCBP1/2 depletion to repress this splicing reaction (Mut-3 and Mut-4 in Supplementary Figure S1). In a reciprocal approach, the native C-rich PPT was replaced with a homopolymeric C_13_ tract (Supplementary Figure S1; Mut-C_13_). This substitution resulted in a decrease in the baseline exon inclusion (48–33%) and fully retained sensitivity to PCBP co-depletion. These data suggested that a C-rich PPT may have a weaker intrinsic splicing activity than the canonical U-rich PPT and that this activity is significantly enhanced by PCBP binding. We conclude from these studies that the PCBP dependence of exon 5 splicing within the CDK2 transcript reflects the C-rich composition of its PPT.

### The PCBP1 isoform has a major impact on CDK2 ex5 splicing

**Figure 2.**
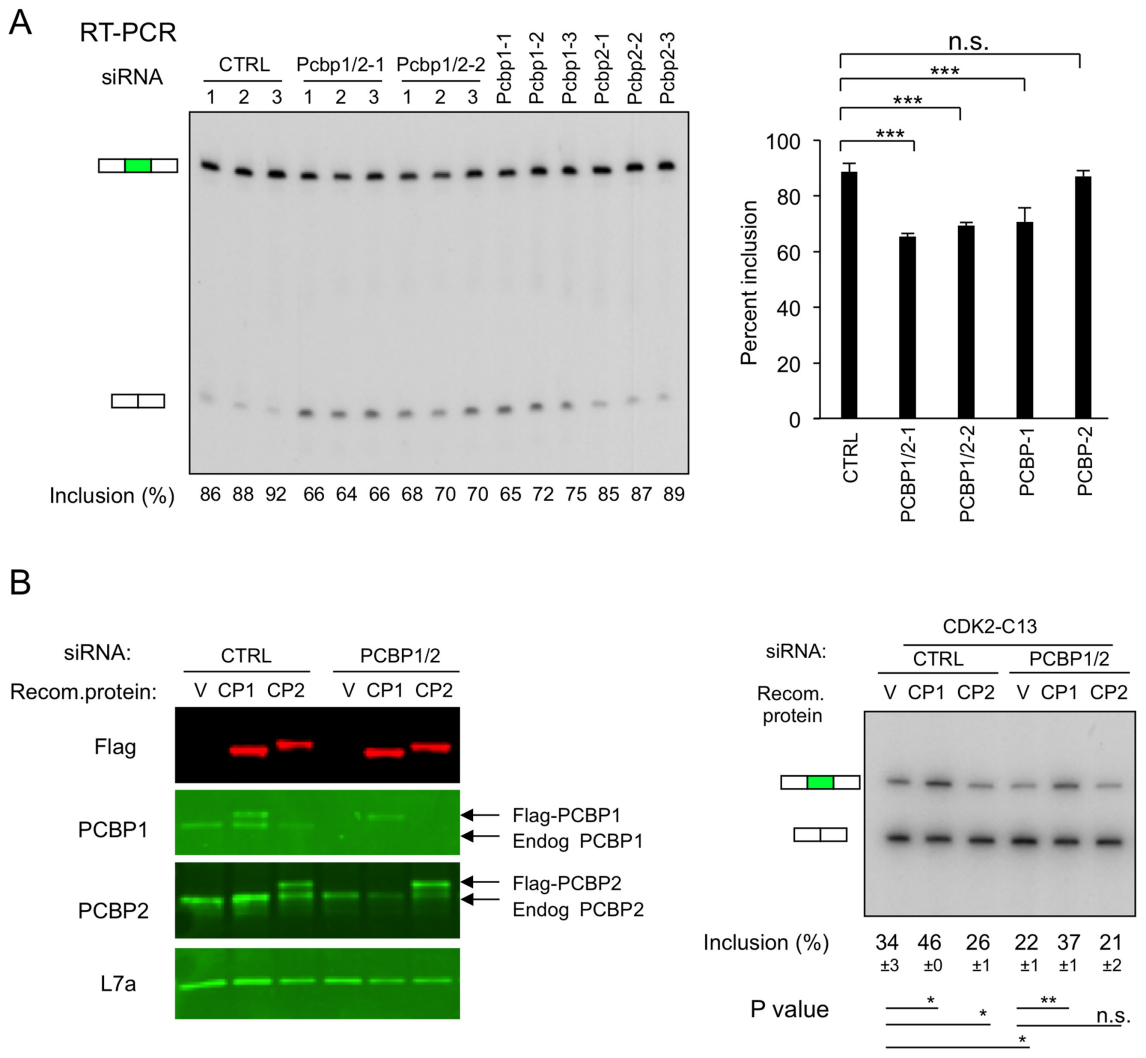
Impacts of the two major PCBP isoforms, PCBP1 and PCBP2, on CDK2 ex5 splicing. (**A**) Impact of isoform-specific depletions of PCBP1 and PCBP2 proteins on splicing of exon 5 in the endogenous CDK2 transcript. K562 cells were individually transfected with 2 distinct siRNAs that co-target PCBP1 and PCBP2 mRNAs (siRNA PCBP1/2-1, PCBP1/2-2 as described (19)). The study was done in biological triplicate or with three distinct siRNAs that selectively target PCBP1 (PCBP1-1, PCBP1-2, PCBP1-3), or PCBP2 (PCBP2-1, PCBP2-2, PCBP2-3). **Left:** Impact of isoform-specific PCBP depletions on cassette exon 5 splicing of the endogenous CDK2 transcript assessed by RT-PCR. **Right:** Quantification and statistics of the cassette exon 5 splicing data. Summary of the splicing inclusion values are shown on the histogram to the right. ^∗∗∗^*P* < 0.001, n.s.: not significant. (**B**) Impacts of PCBP1 and PCBP2 depletion/repletion on CKD2 ex5 mingene splicing. The minigene (CDK2-C_13_) plasmid was co-transfected into K562 cells along with a control siRNA (CTRL) or a siRNA co-targeting PCBP1/2 (siRNA PCBP1/2-2 as in (19)) and/or with plasmids expressing recombinant Flag-tagged PCBP1 or PCBP2 (encoded by siRNA-immune mRNAs). The experiment was carried out twice (*n* = 2). **Left:** Western blot analysis of protein expression. Labeling of lanes: V (empty vector), CP1 (Flag-PCBP1 expression vector), and CP2 (Flag-PCBP2 expression vector). Western blot assay confirmed siRNA-mediated depletion of endogenous PCBP1 or PCBP2 and expression of the recombinant Flag-PCBP proteins. **Right:** Impacts of depletion and repletion on CDK2 ex5 minigene splicing. The mean level of exon 5 inclusion (±SD) in each transfection is displayed below the corresponding lane (*n* = 2). ^∗^*P* < 0.05, ^∗∗^*P* < 0.01, n.s.: not significant.

The two PCBP isoforms, PCBP1 and PCBP2, are conserved in their triple KH domain structures (11,12,21,22), share a strong binding preference for C-rich motifs (10), and have extensive functional overlap in an array of post-transcriptional controls (12). However, it has been demonstrated that these two isoforms can exert distinct functions in a subset of defined model systems (13,23–25). For example, PCBP1 and PCBP2 contribute equally to alternative splicing of the CTTN (exon 11) transcript while PCBP1 is selectively involved in the enhancement of the TARS2 (exon 14) alternative splicing (13). To further explore the respective impacts of the two PCBPs on splicing of CDK2 ex5, they were individually depleted using three distinct siRNAs for each isoform (13). Remarkably, the negative impact of PCBP1 depletion on CDK2 ex5 splicing was equivalent to that generated by PCBP1/2 co-depletion while the selective depletion of PCBP2 had a marginal (non significant) impact on this pathway (Figure 2A). These data lead us to conclude that the PCBP1 isoform plays the major role in PCBP enhancement of CDK2 ex5 splicing.

The isoform specificity of PCBP1 actions on CDK2 ex5 splicing was further assessed by a depletion/repletion study. siRNA-mediated PCBP1/2 co-depletion was accompanied by expression of recombinant Flag-tagged PCBP1or PCBP2 (expressed from siRNA ‘immune’ mRNAs; see Methods) in cells expressing the PCBP-sensitive minigene transcript target, CDK2-C_13_ (diagramed in Figure 1 and Supplementary Figure S1). Co-depletion of endogenous PCBP1/2 and the expression of the Flag-tagged PCBP1 and PCBP2 proteins were confirmed by Western blot (Figure 2B). PCBP1/2 co-depletion had the expected negative impact on exon 5 splicing (34% → 22%) (Figure 2B) and co-expression of Flag-PCBP1 fully restored exon retention level (22% → 37%). In contrast, Flag-PCBP2 expression failed to reverse the PCBP1/2 co-depletion effect. Consistent with its positive role in exon inclusion, expression of Flag-PCBP1 in the control siRNA sample also increased the baseline level of exon 5 inclusion (34% → 46%). Repetition of the minigene depletion/repletion assay on CDK2-WT minigene or CDK2-C26 minigene substrates revealed the progressive decrease in baseline splicing activity as the C-content of the PPT increases (WT > C_13_ > C_26_) and enhancement of the splicing activity of all three PPTs by PCBP (Supplementary Figure S2). These minigene studies lead us to conclude that the enhancement of CDK2 ex5 splicing by PCBPs is predominantly dependent on PCBP1 actions and that the function of PCBP1 is to enhance the activity of an intrinsically weak, C-rich, splice acceptor (also, see below).

### CDK2 ex5 splicing is impacted by a second polypyrimidine tract binding protein, PTB, but not the closely related hnRNPK and RBM39 RBPs

**Figure 3.**
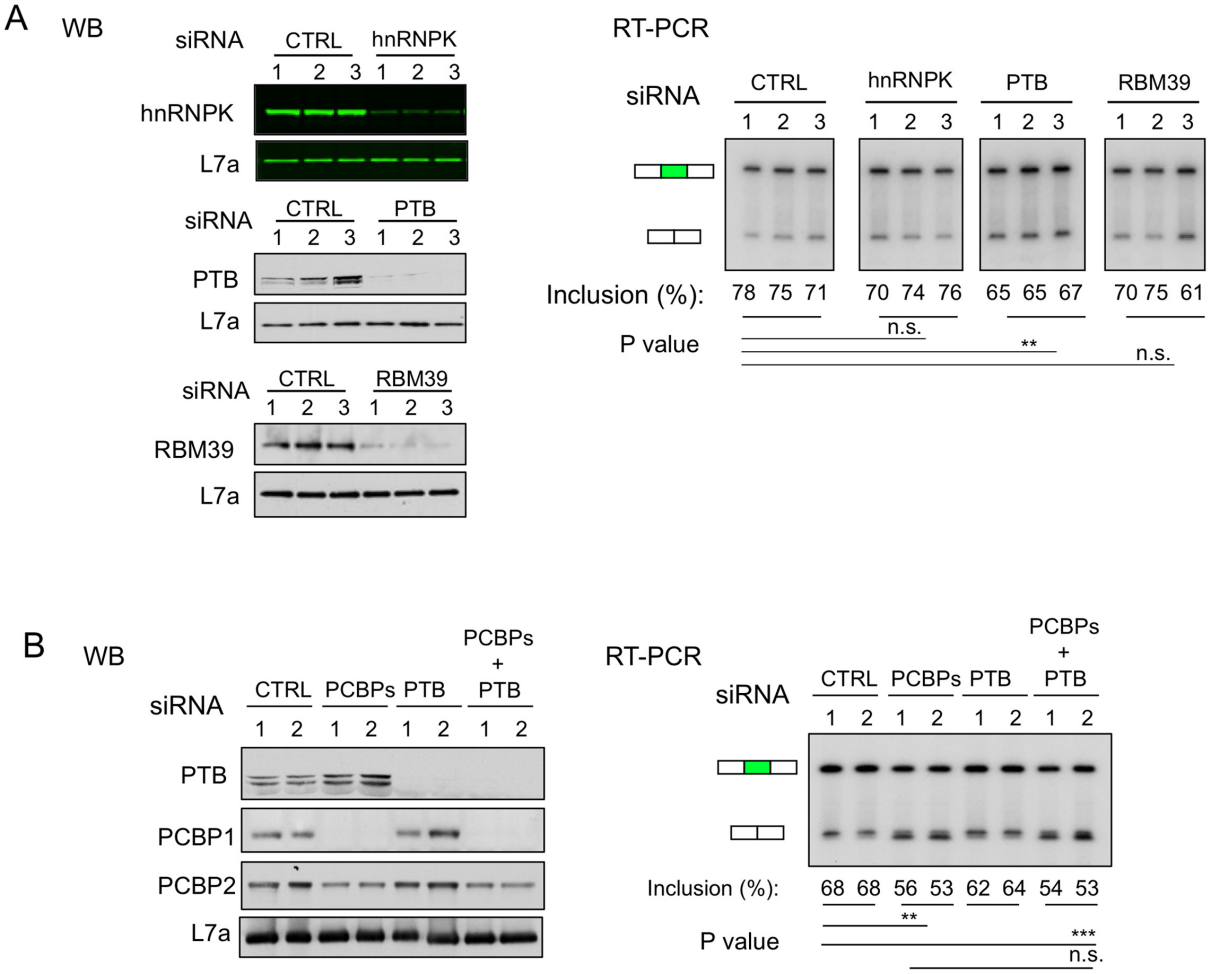
Impact of three additional polypyrimidine binding proteins, hnRNPK, PTB, and RBM39 on CDK2 ex5 splicing. (**A**) Impact of targeted depletions hnRNPK, PTB, or RBM39 on CDK ex5 splicing. K562 cells were individually transfected with 3 distinct siRNAs targeting the mRNAs that encode each of the indicated proteins. **Left:** Depletions of individual proteins were confirmed by WB. Ribosomal protein L7a serves as a loading control. **Right:** The impact of targeted depletions on CDK2 ex5 splicing. Quantification and statistics of the gel are shown at the bottom of the corresponding lanes. n = 3. ^∗∗^*P* < 0.01, n.s.: not significant. (**B**) Impact of dual depletion of PCBPs and PTB on CDK2 ex5 splicing. K562 cells were transfected individually with PCBPs siRNA (siRNA PCBP1/2-2), or PTB siRNA cocktail (mixture of the 3 PTB siRNAs used in Figure 3A), or combination of PCBPs and PTB siRNAs, along with negative control (CTRL), in biological replicates (*n* = 2). **Left:** Western blot confirmations of efficient targeted protein depletions. **Right:** Impact of targeted depletions on CDK2 ex5 splicing as assessed by RT-PCR. Quantification and statistics of the gel were shown at the bottom. ^∗∗^*P* < 0.01, ^∗∗∗^*P* < 0.001, n.s.: not significant. *n* = 2.

PCBP1 and PCBP2 constitute a subgroup of structurally and functionally related RBPs that share varying levels of pyrimidine-binding specificity (12). These RBPs have been linked to both positive and negative impacts on splicing and their functions are mediated primarily *via* associations with the splice acceptor PPT (13). The intrinsically weak activity of the C-rich CDK2 ex5 PPT is subject to regulation (enhancement) by PCBPs when tested both in the context of the native transcript (Figure 2A and (13)) and as a minigene (Figure 1C). Direct comparisons of the PCBP1 and PCBP2 demonstrated a predominant activity of the PCBP1 isoform in CDK2 ex5 splicing control (Figure 2). Furthermore, our prior studies demonstrated that the activity of the C-rich PPT of CDK2 ex5 is not impacted by selective depletion of the canonical PPT binding protein, U2AF65 (13). To further define the specificity of CDK2 ex5 splicing control, we assessed the impact of each of three additional PPT binding proteins on this pathway (Figure 3A). hnRNPK is closely related to the PCBPs with a conserved triple KH domain structure. This RNA binding protein shares a strong preference for polyC binding and has been implicated in modulation of AS pathways (26). PTB is a ubiquitously expressed and intensively investigated splice regulatory protein composed of four RRM motifs (27), it has a defined binding specificity for polypyrimidine tracts containing C bases and can impact on splicing either positively or negatively (6,28). RBM 39 is a U2AF65 paralog (29,30) that has been shown in several cases to interact with PCBP1 and regulate alternative splicing (31,32). A set of three distinct siRNAs was used to selectively deplete each target protein. The impact of each depletion on CDK2 ex5 splicing was assessed. Depletion of both hnRNPK and RBP39 had no significant effect on CDK2 ex5 splicing. In contrast, depletion of PTB caused a small, but significant decrease in exon 5 inclusion (Figure 3A, RT-PCR; also see below). These data suggest that the AS of CDK2 ex5 reflects the specific actions of PCBP1 and to a lesser degree PTB. The lack of impact of the polypyrimidine binding proteins hnRNPK, RBM39, as well as PCBP2 (Figure 2), highlights the specificity of these actions.

To directly compare the activities of PCBPs and PTB on CDK2 ex5 AS, these proteins were depleted either individually or in combination in K562 cells. Depletions were confirmed by Western blot (Figure 3B). The impact of PCBP1/2 depletion on this AS event was more marked than that of PTB (Figure 3B, RT-PCR). Furthermore, the addition of PTB depletion to PCBP depletion failed to accentuate the impact of PCBP depletion alone (Figure 3B). These data lead us to conclude that PCBPs are the major *trans*-acting enhancer of CDK2 ex5 splicing in this experimental system.

### PCBP1 acts independently of U2AF65 in the enhancement of CDK2 ex5 splicing

**Figure 4.**
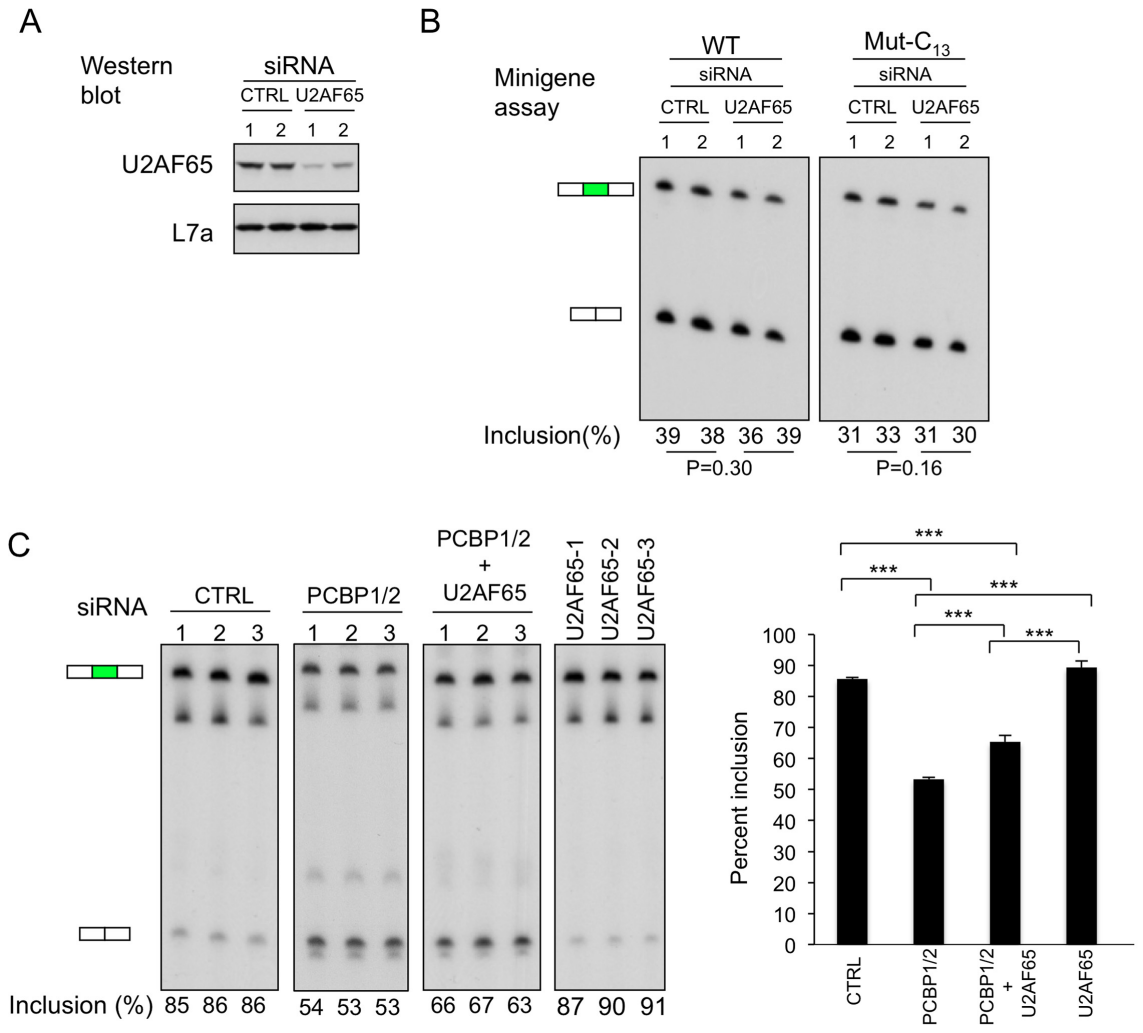
Relative impacts of PCBPs and U2AF65 on CDK2 ex5 splicing. (**A**) Targeted depletion of U2AF65 from K562 cells. The negative control siRNA (Invitrogen, medium GC content) or U2AF65 siRNA (HSS117616, Invitrogen) were transfected into K562 cells in biological duplicates and the level of U2AF65 protein was assessed at 3 days post transfection by Western blot. Loading control corresponds to the ribosomal protein, L7a. (**B**) U2AF65 depletion fails to significantly impact on CDK2 ex5 minigene splicing. Two CDK2 minigenes, one with the wild type PPT and one with a C_13_ substitution in the PPT (as in Figure 1B) were co-transfected along with negative control siRNA (Invitrogen, medium GC content) or U2AF65 siRNA (HSS117616, Invitrogen) in biological duplicates. The level of exon 5 inclusion 3 days post transfection is shown below the corresponding lanes of the gel. *P* values is shown below the gel. (**C**) Specific impact of PCBP versus U2AF65 on CDK2 ex5 splicing. U2AF65 (3 distinct siRNAs) and PCBPs (siRNA PCBP1/2-1, see Figure 2A) were depleted or co-depleted (siRNA PCBP1/2-1 and PTB siRNA HSS117616, Invitrogen) in K562 cells in biological triplicates and their impacts were assessed on exon 5 splicing of the native CDK2 transcript by RT-PCR. *n* = 2. **Left:** RT/PCR analysis of exon 5 inclusion. The positions of the exon 5 inclusion and exclusion bands are indicated to the left of the gel. Quantification of exon 5 inclusion is indicated below the respective lanes. **Right:** The splicing data are plotted on the adjacent histogram. ^∗∗∗^*P* < 0.001. *n* = 3.

The canonical U-rich PPT is bound by U2AF65 in the initial stages of splice site recognition in the great majority of splice acceptors (2). We have demonstrated that targeted depletion of PCBPs has a negative impact on CDK2 ex5 splicing while depletion of U2AF65 fails to impact this pathway (13). Based on these data, generated in the context of the native CDK2 transcript in K562 cells, we proposed a model in which PCBP1 takes the place of U2AF65 in activating splice acceptor activity at a C-rich PPT (13). To facilitate further characterization of the inter-relationships of PCBP and U2AF65 actions, we transfected K562 cells with CDK2 ex5 minigenes in which the PPT was WT or was replaced with a C_13_ track (WT and Mut-C_13_; as in Figure 1B) along with a siRNA targeting U2AF65. Depletion of U2AF65 (Figure 4A) had no appreciable impact on CDK2 ex5 splicing from either PCBP-responsive minigene transcript (Figure 4B). These data support the model in which PCBPs interact with the C-rich PPT to enhance splice acceptor activity and that this enhancement is independent of U2AF65 actions.

As a further test on the interactions and interrelationship of PCBP and U2AF65 actions, we assessed the impact of their combined depletion on exon 5 splicing from the native CDK2 transcript (Figure 4C). The marginal positive impact of individual depletion of U2AF65 on exon 5 inclusion contrasted with the pronounced decrease of exon 5 inclusion in response to PCBP1/2 depletion. The combined depletion of U2AF65 along with PCBP1/2 co-depletion resulted in an increased inclusion of exon 5 over that of PCBP1/2 co-depletion alone, but still at levels well below that in the control setting (Figure 4C). These data suggest that U2AF65 may have a small negative impact on the C-rich PPT controlling CDK2 ex5 splicing while the impact of PCBPs at this site is strongly enhancing. These studies further support the model in which PCBP specifically enhances splicing of CDK2 ex5 and that its activity is independent of, and possibly opposite to, the actions of U2AF65 at this site.

### CDK2 ex5 splicing impacts on CDK2 protein expression and cell cycle kinetics

**Figure 5.**
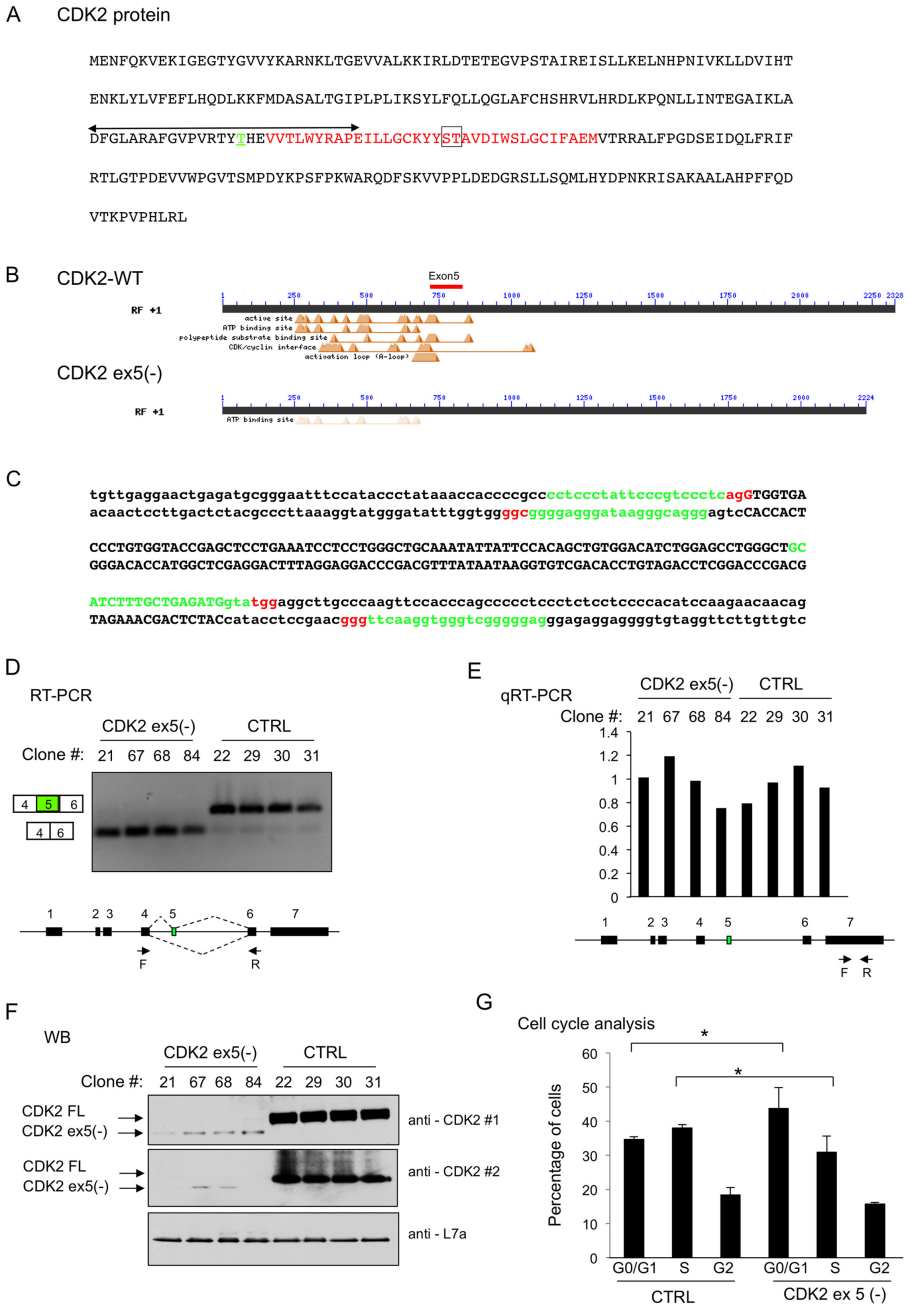
CDK2 ex5 encodes a protein segment critical to CDK2 function. (**A**) Human CDK2 protein primary structure. The region encoded by exon 5 is in red font; the CDK2 ‘activation domain’ is indicated by double headed arrow; T160 phosphorylation site critical to CDK2 action is in green font and underlined; the ST(181–182) dipeptide critical for CDK/cyclin interface is boxed. (**B**) Conserved Domain (CD) analysis (http://www.ncbi.nlm.nih.gov/cdd) of CDK2 (CDK2-WT) and the protein encoded by the CDK2 ex5(–) mRNA. The position occupied by the segment of CDK2 encoded by exon 5 is shown as a red bar. The CD analysis of the CDK2 ex5(-), shown on the second line, reveals a predicted loss of the major functional domains. (**C**) sgRNAs designed to ablate exon 5 region from the CDK2 locus via directed Cas9 cleavages. The double-stranded sequence of the genomic DNA encoding exon 5 (upper case) and surrounding intron sequences (lower case) is shown. The positions of the four sgRNAs used in the study to target Cas9 cleavage are highlighted in green and the corresponding PAM sequences are shown in red. (**D**) Generation of K562 cell clones that exclusively express the CDK2 ex5(-) mRNA. RT/PCR analysis (as in Figure 2) of four WT control K562 cell clones (CTRL) and four clones with CDK2 exon 5 exclusion *via* direct mutations of the genomic loci (CDK2 ex5(–)). The designation of each clone is indicated above the corresponding lane. Primer positions are shown. (**E**) qRT-PCR analysis. CDK2 mRNA in the four control (CTRL) and four CDK2 ex5(–) clones was quantified by real-time PCR (primers shown in diagram below graph). The average of CDK2 levels in the four control clones was arbitrarily set as 1. Overall, total CDK2 mRNA levels are comparable in these two groups. (**F**) Western blot analysis of CDK2 ex5(–) K562 clones. The identity of each cell clone is shown above the corresponding lane. The positions of the full-length CDK2 (CDK2 FL) protein, the CDK2 ex5(–), and the loading control L7a are indicated. CDK2#1 and CDK2#2 are two distinct CDK2 primary antibodies. (**G**) Impact of CDK2 ex5 deletion on cell cycle kinetics. FACS analysis was carried out on cells following propidium iodide (PI) staining of three control cell clones (CTRL) and 3 CDK2 ex5(-) cell clones. The percentages of cells in each of the identified phases (G0/G1, S and G2/M) were quantified and were plotted in the histogram. Significance comparisons are as indicated (*n* = 3). ^∗^*P* < 0.05.

Our data demonstrate that the PCBPs, and more specifically the PCBP1 isoform, enhance CDK2 ex5 splicing by acting at a C-rich PPT 5′ to exon 5 splice acceptor. We next explored how the inclusion/exclusion of exon 5 impacts on CDK2 protein expression and function. The full length (i.e. exon 5(+)) CDK2 mRNA encodes a 297 amino acid protein (33,34) (Figure 5A). This CDK2 protein must be phosphorylated at residue T160 to activate its cell cycle regulation functions (14). This phosporylated-residue is located within an ‘activation’ segment (aa 145–172; double headed arrow in Figure 5A) that is essential to CDK2 interactions with cyclins E and A and to recognition of its protein phosphorylation substrates (35,36). The exon 5 encoded segment (aa 163–196; red font in Figure 5A) overlaps with the activation segment, is in close proximity to the T160 phosphorylation site, and contains residues (amino acid 181–182) that contribute to the CDK/cyclin interaction interface (35,36). Thus, the loss of exon 5 would be predicted to trigger a major disruption of multiple protein segments and determinants critical to CDK2 functions in cell cycle control. The potential impacts of this disruption are highlighted by the structure/function prediction as shown in Figure 5B (Conserved Domain (CD, http://www.ncbi.nlm.nih.gov/cdd)) (35,36).

The impact of exon 5 deletion on CDK2 protein expression was directly assessed by targeted mutation of the CDK2 locus in the K562 genome. Four sgRNAs flanking exon 5 were designed to overlap with, or be in close proximity to, the exon 5 splice donor and acceptor splice junctions (Figure 5C). These four sgRNA vectors with a Cas9 expression cassette were transfected into K562 cells. Transfected cells were antibiotic selected, cloned by terminal dilution, expanded, and sequenced across the targeted CDK2 locus. Cell lines with mutations that were predicted to ablate exon 5 and/or eliminate its splicing were identified and expanded for analysis. The structures of the CDK2 genes in the four control clones were determined by sequencing through the targeted region. Each of the four CDK2 exon 5 (–) clones were documented to contain mutations at both CDK2 alleles that eliminate exon 5 inclusion (see Methods for details). This set of clones were referred to as CKD2 ex5(–). The complete absence of exon 5 from the CDK2 mRNA was confirmed in all four of these cell clones (Figure 5D).

The impact of deletion of exon 5 on CDK2 expression was next assessed. Sequence analysis of CDK2 mRNA from the exon 5(–) clones confirmed that the targeted deletion of the 102 bp exon 5 did not alter the open reading frame of the CDK2 mRNA. Real-time RT-PCR analysis further demonstrated that exon 5 deletion had no significant impact on steady state levels of CDK2 mRNA (Figure 5E). The western blot of cell extracts from each of the four clones with exon 5 inactivation confirmed the expected loss of full length CDK2 protein. This immunoblot further revealed a novel CDK2-reactive band that corresponded in size to the predicted molecular weight of CDK2 lacking exon 5 (four KD shortened) (Figure 5F). The intensity of this band was markedly weaker than the band representing the full length CDK2 protein in the WT cells. These findings were reproduced by Western blots using a second independent CDK2 antibody (antibody #2; Figure 5F). These data lead us to conclude that the protein segment of CDK2 encoded by exon 5 is critical to the steady state expression of CDK2 protein.

CDK2 is an intensively studied component of cell cycle regulation (15,16,35). Therefore, we next determined the functional consequence of exclusion of CDK2 ex5 on the cell cycle regulation. Cells from three control WT clones and three CDK2 ex5(–) clones were assayed for cell cycle distribution by FACS analysis (Figure 5G). This direct comparison revealed a significant enrichment in the G0/G1 phase for the CDK2 ex5(–) cells *vs* the WT cells with a reciprocal depletion of the proportion of cells in S phase. These data are consistent with the known function of CDK2 to regulate the G0/G1 to S phase transition in K562 cells (37). We conclude from these data that exclusion of exon 5 from CDK2 mRNA results in a dramatic decrease in CDK2 protein expression with a corresponding impact on the G0/G1 to S phase transition.

**Figure 6.**
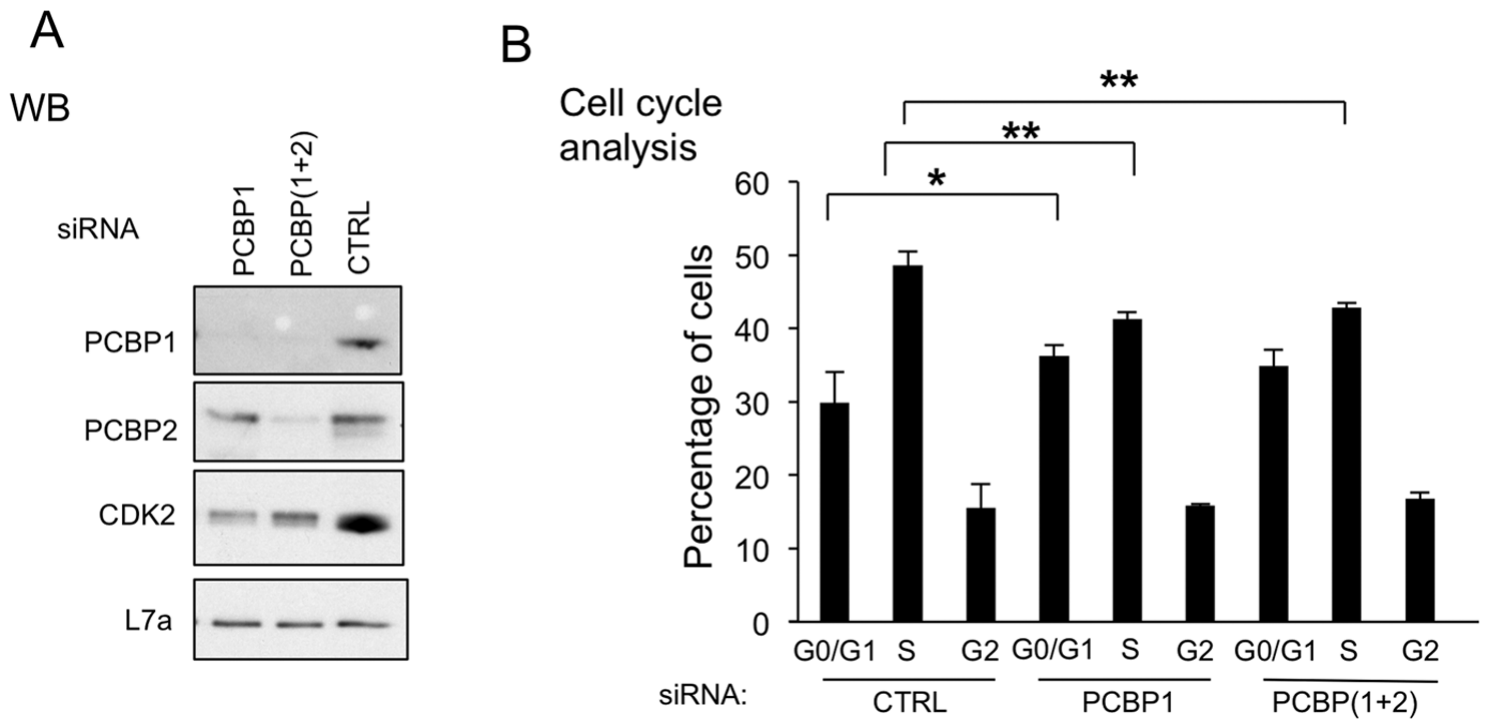
PCBPs control CDK2 protein level and cell cycle kinetics. (**A**) PCBP1 or combined PCBP1 and PCBP2 (PCBP(1+2)) were depleted by the indicated siRNAs. A cocktail of three distinct PCBP1 siRNAs, or the combination of PCBP1 siRNA cocktail and PCBP2 siRNA cocktail were transfected into K562 cells in biological triplicates, respectively. This western blot assesses the levels of PCBP1, PCBP2, and CDK2 protein expression in the indicated cell transfections. Loading control; L7a. (**B**) Cell cycle analysis of samples shown in A. Analysis as described in Figure 5G, above. ^∗^*P* < 0.05, ^∗∗^*P* < 0.01

To further confirm that PCBP proteins can directly regulate CDK2 protein level and cell cycle progression, we depleted K562 cells of PCBP1 or co-depleted PCBP1 and PCBP2 and demonstrated that these depletions resulted in the predicted decrease in CDK2 protein (Figure 6A) and the same shift in the relative distribution of cells in G1/Go and S phase (Figure 6B) as was seen with the direct deletion of exon 5 *via* genomic editing (Figure 5G). Taken together, these data confirm that PCBPs, and specially PCBP1, regulate the AS of CDK2 exon 5 with a consequent impact on expression of CDK2 protein and cell cycle kinetics.

### Comparison of CDK2 splicing in mouse and human cells suggests that alternative splicing of exon 5 has been acquired recently in mammalian evolution

**Figure 7.**
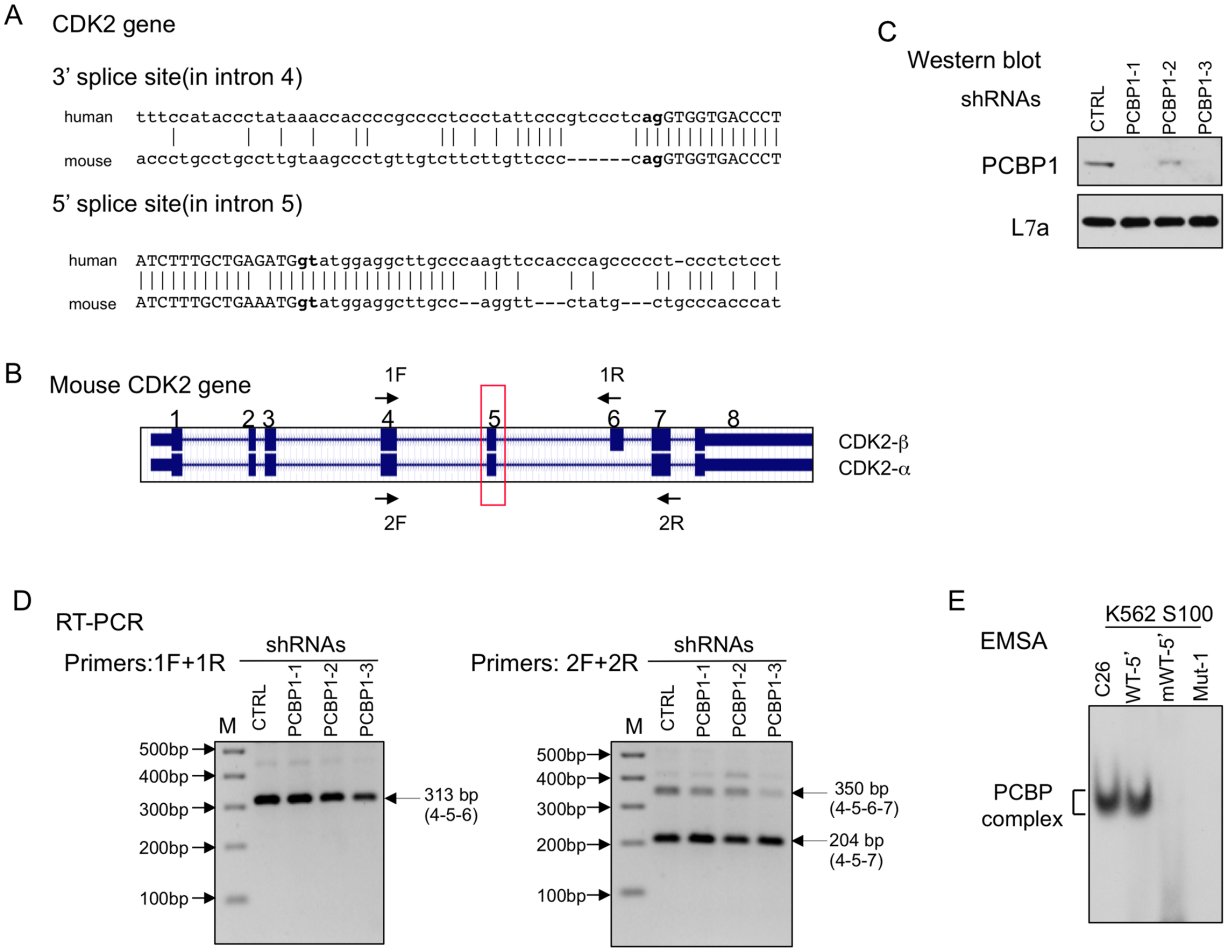
Alternative splicing of CDK2 ex5 is specific to human vs mouse. (**A**) Comparison of intronic sequences flanking CDK2 ex5 in humans *vs* mouse. Intronic sequences are in lower case and exonic sequences are in upper case. Gaps and identity in this maximal alignment are indicated by dashes and connecting lines, respectively. The acceptor dinucleotide, **ag**, and the splice donor dinucleotide, **gt**, are bolded. (**B**) Two CDK2 isoforms are generated in the mouse *via* alternative splicing of exon 6 while splicing of exon 5 in the mouse (boxed in red) is constitutive. Sequence corresponding to exon 6 sequences can be identified in the human CDK2 gene within intron 5, but this region is not spliced into the human CDK2 mRNA. The two primer sets used for splice analysis (RT/PCR, in D) is indicated by the F (forward) and R (reverse) arrows. (**C**) Depletion of mPCBP1 in mouse erythroleukemia (MEL) cells. Three independent shRNAs targeting PCBP1 mRNA (shRNAs PCBP1-1, 1-2, 1-3) and a control shRNA (CTRL) were used in this study. The efficiency of PCBP1 depletion was confirmed by Western blot with antibodies to PCBP1 or loading control L7a. (**D**) PCBP depletion fails to impact on CDK2 exon 5 or 6 splicing in mouse cells. RT-PCR analyses of RNA isolated from PCBP1 depleted *vs* Control cells: **Left:** RT-PCR with primer set 1F (in exon 4) and 1R (in exon 6). The 313 bp PCR product corresponds to exons 4–5–6. This assay fails to detect exon 5 skipping (predicted to be 211 bp). **Right**: RT-PCR with primer set 2F (in exon 4) and 2R (in exon 7). This RT-PCR generate only two RNA species; the 350 bp product corresponds to CDK2β (exons 4–5–6–7) and the 204bp product corresponds to CDK2α (exons 4–5–7). This assay fails to detect evidence of exon 5 skipping (predicted to be 248 bp). The identities of all three RT-PCR products were confirmed by Sanger sequencing. M: 100 bp DNA ladder. (**E**) PCBP binds to the exon 5 splice acceptor region of the human, but not the mouse, CDK2 transcript. PCBP binding to the human (WT-5′) and mouse (mWT-5′) CDK2 exon 5 PPT splice sites was directly compared *in vitro* by RNA EMSA (as in Figure 1D). The C_26_ homoribopolymer served as a positive control and Mut-1 RNA (Figure 1B) as a negative control for the formation and migration of the PCBP/RNA complex.

The preceding studies demonstrate PCBP-controlled alternative splicing of CDK2 ex5 in a human hematopoetic cell line (K562). This splicing pathway has a dramatic impact on CDK2 protein expression with a corresponding impact on cell cycle regulation (Figure 5). Consistent with its pivotal role in CDK2 protein expression and function, exon 5 encodes a protein segment that is 100% conserved in the CDK2 protein from zebra fish to humans. It is of note in this context that the C-rich intronic sequences flanking exon 5 is highly conserved among primate species but diverges in subprimate mammalian species (UCSC genome browser conservation panel, data not shown). This divergence is highlighted by a direct comparison of the CDK2 exon 5 splice acceptor of the human and mouse; visual comparison reveals that the PPT is substantially more C-rich in humans than in the mouse due to a segmental deletion and multiple C → T transitions in the mouse genome (Figure 7A). Public data (UCSC Genome Browser on Mouse December 2011, GRCm38/mm10 Assembly) further reveals that CDK2 ex5 splicing in the mouse transcript is constitutive (always included) while the adjacent exon (exon 6) is alternatively spliced. The alternative splicing of mouse CDK2 exon 6 generates two major protein isoforms: one lacking exon 6 encoded structures (CDK2-α; same structure as human full length CDK2) and one including exon 6 (CDK2β) (Figure 7B) (38,39). These data reveal a clear difference in the splicing control within the human and mouse CDK2 transcripts; the alternative splicing of exon 5 in the human CDK2 transcript is linked to the accrued C-rich composition of its PPT.

To specifically confirm that mouse CDK2 ex5 splicing is constitutive, we assessed mCDK2 splicing in mouse erythroleukemia cells (MEL). Targeted amplification of CDK2 mRNA was performed between exons 4 and 6 and between exons 4 and 7. Consistent with prior reports (38,39), both sets of studies confirmed constitutive exon 5 inclusion (Figure 7B and D). Furthermore, the inclusion of exon 5 in the mouse CDK2 mRNA was not impacted by the depletion of PCBPs from the MEL cells (Figure 7C, D). The lack of impact of PCBPs on mCDK2 ex5 splicing was consistent with an inability of PCBPs to bind to the PPT 5′ to mCDK2 ex5 as assessed by RNA-EMSA (Figure 7E). These data lead us to conclude that in the mouse, exon 5 is flanked by a canonical PPT that is not recognized by PCBP. In humans the PPT has evolved to a C-rich structure that renders hCDK2 ex5 splicing sensitive to PCBP. Thus, the function(s) of PCBP as a splicing regulator of human CDK2 ex5 was likely acquired recently during mammalian evolution in the primate lineage. Such recently acquired control may serve to fine-tune cell cycle control in a variety of settings (see Discussion, below).

## DISCUSSION

CDK2 has been intensively studied as a critical regulator of cell cycle kinetics and cell proliferation (17). CDK2 partners with cyclin E to drive the cell though the G1/S transition and then partners with A-type cyclins to promote progression through S phase (17). Targeted inactivation of CDK2 demonstrates an essential role in meiosis (15,16). An essential role for CDK2 in mitotic cell division is less well established, most likely due to molecular redundancy and compensation by related CDKs (15,16). De-regulation of CDK2 activity has been linked to functional impacts in a variety of cancers (17,40–44). Besides its defined functions in cell cycle control, CDK2 has also been linked to key regulatory steps in the pathway of cell senescence (45,46). Active searches are ongoing to identify and test the efficacy of CDK2 inhibitors in a number of clinical settings (17,40). Thus understanding the pathways involved in the control of CDK2 expression has clear relevance in both normal and pathologic setting.

The vast majority of mammalian gene transcripts undergo alternative splicing (47). This post-transcriptional control pathway is a powerful generator of transcriptome diversity, greatly increasing the number of proteins that can be generated from a fixed genome. The plasticity of alternative splicing can further shuffle the mix of protein isoforms to respond rapidly to a variety of developmental/differentiation cues and external environmental alterations. Prior studies have revealed that alternative splicing of cell cycle related gene transcripts can impact on a variety of cellular functions and pathologic states (48–50).

Here we focus on the expression of the cell cycle dependent kinase, CDK2. In a prior study we described a screen for shifts in cassette exon splicing that are under the control of the PCBPs in the mammalian transcriptome. In that study we observed that CDK2 exon 5 splicing is markedly decreased (87% → 48%) in cells acutely co-depleted of PCBP1/2 (13). We now extend these initial findings by exploring the *cis* and *trans* acting determinants of this CDK2 post-transcriptional control. Comparison of PCBP1 and PCBP2 actions demonstrate that the major impact of the PCBP1/2 co-depletion on CDK2 ex5 splicing reflects the activity of the PCBP1 isoform. In contrast, the impact of PCBP2 depletion was marginal at best (Figure 2). A series of depletion/repletion studies further revealed that PCBP1 is both necessary and sufficient for enhancing activity of the CDK2 ex5 splice acceptor (Figure 2).

A substantial literature documents that the PCBP1 and PCBP2 isoforms have multiple overlaps in their biologic functions (13,19,51–54). This frequency of overlapping functions is consistent with the highly conserved structures of their triple KH-domain structures. At the level of primary structure, PCBP1 and PCBP2 have an 86% level of overall identity with 93% identity in the sequence within the 3 KH domain regions. We have previously demonstrated that the PCBP1 locus was generated during evolution *via* retrotransposition of a copy of a PCBP2 mRNA isoform (12,21,22). Importantly, PCBP1 and PCBP2 have well documented distinctions in biologic functions. As a prominent example, PCBP1 has a selective role in regulation of the epithelial to mesenchymal transition (55–58). Furthermore, we have demonstrated *in vivo*, using mouse gene ablation studies, that PCBP1 and PCBP2 genes are independently essential to embryonic development (23). It remains unclear whether the distinct timing and mechanisms of the observed embryonic lethalities in the two settings reflect distinct functions of the PCBP1 and PCBP2 proteins and/or distinct temporal and spatial patterns of their relative expression during development.

Regulation of AS is a complex process that involves multiple *cis*-elements and *trans*-acting factors (2). The location of the *cis*-regulatory elements and the identity of the corresponding protein binding partners dictate the outcome of the final mRNA product. For example, the RNA binding protein PTB can act as either an enhancer or repressor of cassette exon splicing depending on the structure and positioning of the RNA elements with which it interacts (6,28). In an initial screen we identified that more than 900 cassette exons were impacted by acute co-depletion of PCBP1/2 in K562 cells (13). We observed a remarkable concordance between the cassette exons that were repressed in these acute depletion studies and the occurrence of a PPT 5′ to the impacted exon that was markedly enriched for C rather than the canonical U composition. In the present study we have explored the specificity of the PCBP control over these C-rich PPT by assessing the impacts of PCBPs with a set of RNA binding proteins with a shared binding specificity for polypyrimidine motifs. We found that acute depletions of hnRNPK, U2AF65, and the U2AF65 paralog, RBM39, had no significant role in this pathway (Figures 3 and 4). In contrast, depletion of PTB triggered an accentuation of exon 5 skipping. This impact of PTB was clearly less robust than that of PCBP1. The observation that co-depletion of PTB along with PCBP1 failed to accentuate the impact of PCBP1 depletion alone argues against a simple additive mechanism. These interactions are more likely to be complex as many of these RNA binding proteins may exist and function in the context of complex multicomponent assemblies (e.g. LASR (59)). The basis for the PTB actions and their distinctions from those of PCBP1 remain to be explored in future studies.

**Figure 8.**
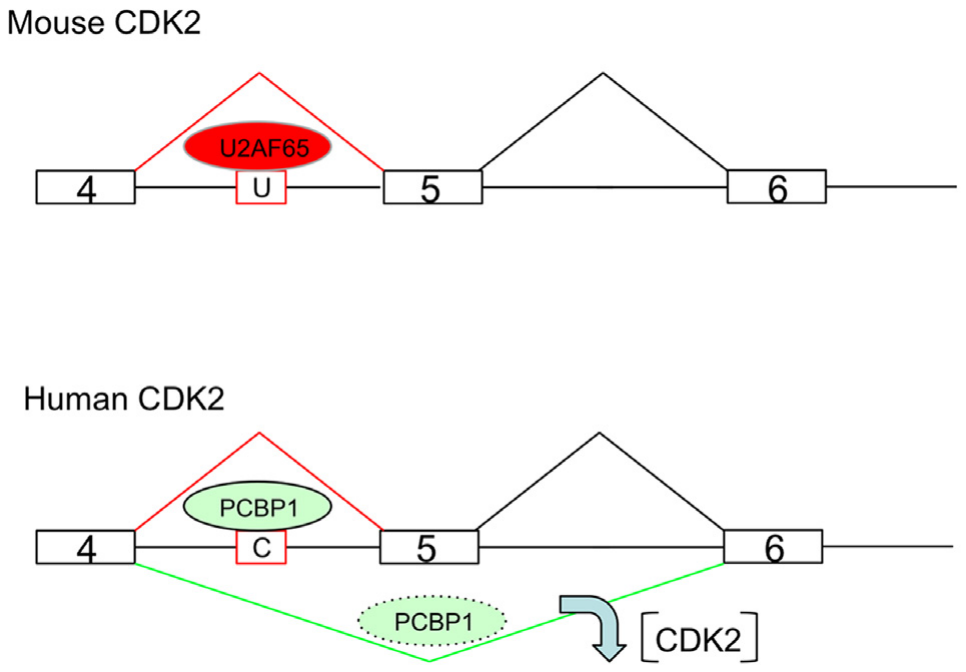
Model: PCBP1 controls exon 5 splicing of the human CKD2 gene transcript *via* interaction with a recently evolved C-rich PPT. The regions of the mouse and human CDK2 genes that encompass exon 5 and its two flanking exons are shown (not drawn to scale). U: U-rich PPT; C: C-rich PPT. U2AF65 and PCBP1 are RNA binding proteins interacting with U-rich PPT, C-rich PPT, respectively. The mouse U-rich PPT interacts with U2AF65, the canonical PPT binding protein, to ensure that mouse CDK2 exon 4 is spliced to exon 5 constitutively. Human C-rich PPT interacts with PCBP1 proteins to regulate the inclusion of exon 5 in the hCDK2 transcript. The efficiency of AS event is likely to be impacted by the PCBP1 availability and activity (level and modification). When PCBP1 is available, it binds to the C-rich PPT resulting in the inclusion of exon 5. When PCBP1 level/activity is reduced in the cells, exon 5 will be skipped and CDK2 protein expression will be markedly diminished (downward arrow). By this mechanism, the cell cycle can be modulated in response to external or internal cues, through alterations CDK2 expression.

Given the importance of CDK2 to a number of biologic functions and the highly conserved structure of this protein in mammalian species, it is surprising that the mouse and human CDK2 transcripts are under distinct splicing controls. There are two differences that are of particular interest in comparing human and the mouse CDK2 splicing patterns. First, the human CDK2 gene contains seven exons, while mouse CDK2 contains 8 exons (38,39). The DNA segment encoding the ‘extra’ exon in the mouse CDK2 gene, exon 6, exists in human CDK2 DNA sequence within intron 5. Remarkably, this covert exon is not spliced into the human mRNA even though the splicing consensus sequences are well maintained. It has been speculated that this lack of inclusion of exon 6 reflects the presence of a strong lariat formation site at the human CDK2 exon 6 splicing acceptor that drives direct splicing of human exon 5 of CDK2 to human exon 6, thus skipping the covert exon (38). Mouse CDK2 exon 6 is alternatively retained in the mouse CDK2 mRNAs generating two CDK2 isoforms (see Figure 7B schematic). These two mouse CDK2 isoforms seem to have similar function(s), but with somewhat differential catalytic activity (38,39). Importantly, this pathway of AS is unaffected by PCBP activity. Thus the splicing patterns of the mouse and human CDK2 transcript are clearly distinct, with the later being specifically under PCBP control (Figure 8).

The other difference between human and mouse CDK2 splicing patterns at exon 5 are of direct interest to the current study. Exon 5 of mouse and human CDK2 encode identical protein segments. While the human CDK2 ex5 is alternatively spliced, the splicing of the mouse CDK2 ex5 is constitutive. Based upon the current analysis of exon 5 splicing control, and the importance of the PPT structure, we suggest that this difference relates to the sequence composition of the splice acceptor. The PPT at the human CDK2 ex5 splicing acceptor site is markedly C-rich while that in the mouse gene has multiple C →T transitions and more closely resemble a canonical U-rich PPT site (Figure 7A). This shift in sequence at the PPT results in a selective binding of PCBP to the human splice acceptor (Figure 7E) and the lack of response to PCBP depletion in mouse cells (Figure 7D). Thus, it is likely that the CDK2 ex5 splicing shifted from constitutive to alternative due to evolution in the structure of the exon 5 PPT.

In this report, we demonstrate that PCBP exerts a positive impact on CDK2 expression *via* modulation of cassette exon splicing. It is of interest, in this regard, that we have previously shown that PCBPs also has a positive impact on CDK2 activity by negatively regulating the stability of the mRNA encoding P21, the major inhibitor of CDK2 kinase (60). Our studies further revealed that mRNAs encoding P27 and P57, the other two major CDK2 kinase inhibitors, are also increased upon PCBP1/2 depletion, although the mechanism in these two instances have not been determined. Thus PCBP proteins appear to exert their positive impact on cell cycle regulation by modulating CDK2 activity through multiple mechanisms at different points in the CDK2 regulatory network (Supplementary Figure S3).

Modulation of CDK2 ex5 splicing has a direct impact on expression of CDK2 protein expression (Figure 5). Thus alterations in levels of PCBPs in the cell capable of binding to and enhancing the activity of the C-rich PPT at the CDK2 exon 5 splice acceptor site becomes a critical determinant of cell cycle control. Complex levels of post-translational modifications of the PCBPs impact on their RNA binding activity and nuclear/cytoplasmic compartmentalization. For example, phosphorylation of S-43 residue of PCBP1, which reflects the activity of the TGFβ pathway (55), has a strong negative impact on its RNA binding activity (55,56). PCBPs shuttle between nucleus and cytoplasm (61) and this trafficking is under the control of a novel set of nuclear export and import signals (61), and signaling-dependent phosphorylation of threonine T60 and T127 of PCBP1 can modify its subcellular compartmentalization (31). Such alterations in post-translational modifications and partitioning of PCBP1, could serve to impact on its availability/ability to alter splicing events in response to external environmental cues. This connection is further highlighted by the relationship of PCBP1 RNA-binding activities to levels of cellular nutrients, such as iron and folate (24,25). The level of iron loading may toggle PCBP1 RNA binding activity in a fashion similar to that well documented for the IREBP proteins (62) and can modulate cell cycle regulation through CDK2 (63). Involvement of CDK2 in apoptotic and cell senescence pathways further broadens the implications of its splicing regulation (45,46). Finally, PCBP1 activity can be regulated by dynamic interactions with partner proteins (32,64). Thus the impact of PCBP1 on CDK2 ex5 AS and subsequent CDK2 protein expression can reflect the convergence of multiple and interconnecting cell regulatory pathways that transduce a multitude of biological stimuli. In view of these impacts on basic cellular functions, pathways that control CDK2 expression, once delineated, can help define a subset of pathologic states and identify novel therapeutic targets (17,40). The present study serves to identify a novel pathway that controls levels of CDK2 activity by focusing on the biogenesis of its encoding mRNA.

## Supplementary Material

Supplementary Data
