# Supplementary material for: PolyC-binding proteins enhance expression of the CDK2 cell cycle regulatory protein via alternative splicing
Source: Nucleic Acids Res. 2017 Dec 14;46(4):2030–44. doi: 10.1093/nar/gkx1255 (PMC5829739; doi:10.1093/nar/gkx1255)

Supplemental Figure. 1

CDK2 ex5 minigenes with altered C and U content in the splice acceptor PPT.

A. The CDK2 ex5 along with its contiguous intron sequences (red) was inserted into a minigene vector (as in **Fig. 1).** Base substitutions in the C-rich splice acceptor PPT flanking the CDK2 ex5 splice acceptor are highlighted in blue. In the C_{13} minigene, the PPT was replaced by a homopolymeric C sequence. Each minigene construct was transfected into K562 cells and the transcript was assessed for exon 5 inclusion in the steady state RNA as in panel C.

B. RNA EMSA analyses of mutant splice sites. Mut-3 and Mut-4 disrupt PCBP complex formation. C-13 minigene forms a strong PCBP/RNP complex.

C. Splicing analysis. These three minigene plasmids were co-transfected into K562 cells along with plasmids expressing the PCBP1/2 shRNA or a control shRNA. The splicing efficiency of exon 5 was assessed by RT-PCR after a 3-day incubation. The % inclusion was calculated as in **Methods**.

Supplemental Figure 2

PCBP1 is the major PCBP isoform to enhance inclusion of cassette exons *via* a C-rich splice acceptor site.

A. Co-transfections were performed with minigenes (CDK2-WT or CDK2-26C) and shRNAs as indicated, along with vectors expressing recombinant PCBPs encoded by shRNA-immune mRNAs. These two minigenes were transfected into K562 cells along with PCBP1/2 shRNAs and Flag-tagged PCBPs expression vector (V: empty vector ;

CP1:PCBP1 expression vector; CP2: PCBP2 expression vector. Western blot assay to confirm the siRNA-mediated depletion and recombinant PCBP expression.

B. CDK2-WT minigene splicing was assessed by RT/PCR.

C. CDK2-26C minigene splicing was assessed by RT/PCR.

Supplemental Fig. 3

PCBPs modulate CDK2 activity *via* multiple mechanisms. PCBP1/2 repress the expression of all three CDK2 kinase inhibitors (P21, P27 and P57)((60) and our RNA-seq data) and enhance CDK2 protein expression (**Fig. 5** of this report). Thus, PCBPs can modulate CDK2 activity cell cycle kinetics by coordinately altering levels of its kinase inhibitors and by directly impacting on CDK2 protein.

Supplemental Figure 1

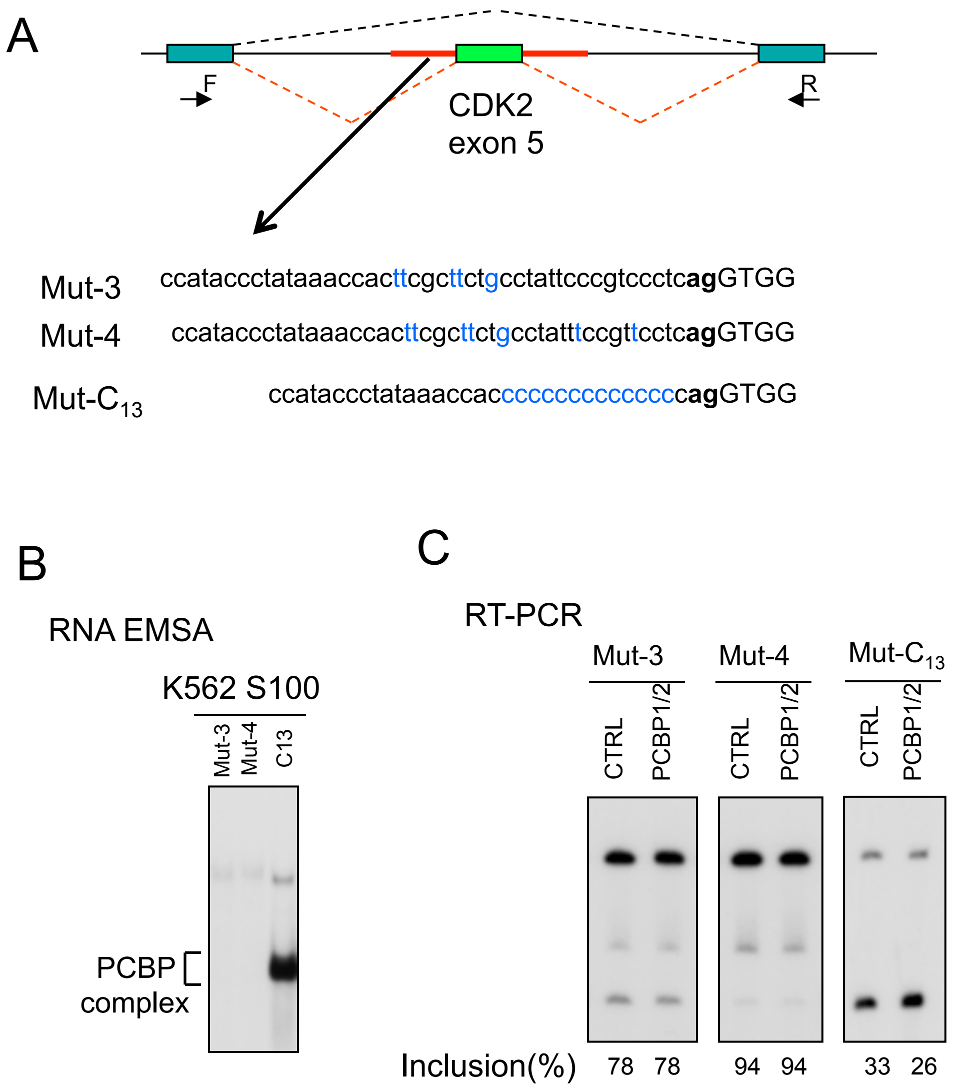

Supplemental Figure 2

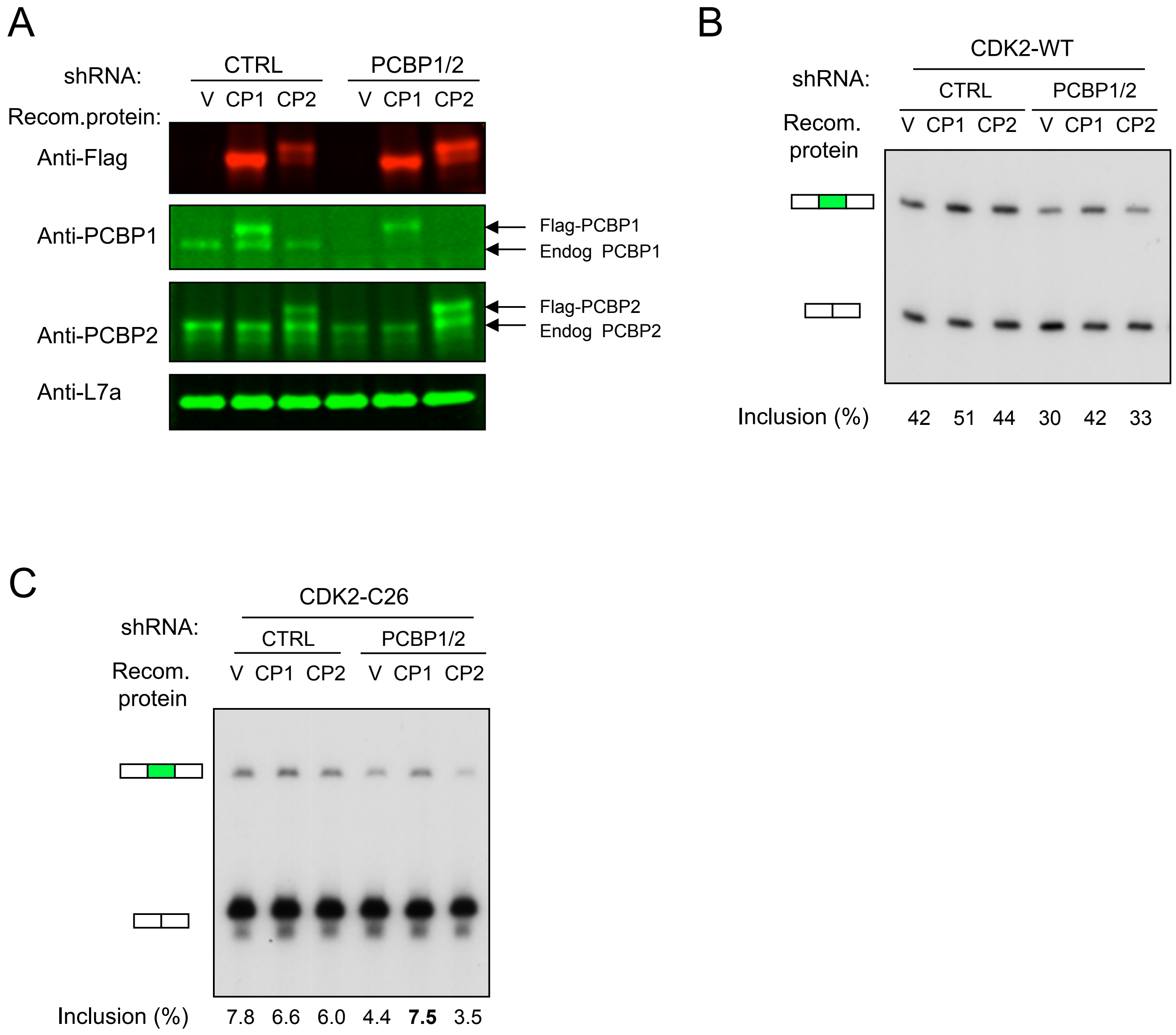

Supplemental Fig. 3

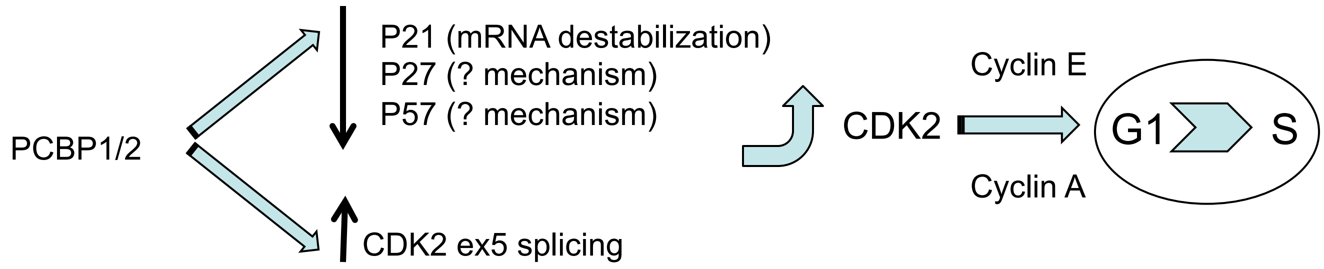

Supplement: Supplementary Data [file gkx1255_supp.pdf]
